# Normality of sagittal spinal alignment parameters reveals evolutionary signals in healthy adults across five countries

**DOI:** 10.1038/s41598-025-19366-z

**Published:** 2025-10-10

**Authors:** Kazuhiro Hasegawa, Kei Watanabe, Masayuki Ohashi, Shun Hatsushikano, Zeeshan M. Sardar, Jean Charles Le Huec, Stephane Bourret, Michael P. Kelly, Hee-Kit Wong, Hwee Weng Dennis Hey, Gabriel Liu, Hend Riahi, Lawrence G. Lenke

**Affiliations:** 1 Niigata Spine Surgery Center, 2-5-22 Nishi-machi, Konan-ku, Niigata, 950-0165 Japan; 2 Division of Orthopedic Surgery, Department of Regenerative and Transplant Medicine, Niigata University Graduate School of Medical and Dental Sciences, Niigata, Japan; 3 VERTEBRA Institut, Bordeaux Nord Aquitaine Polyclinic, Bordeaux, France; 4 Rady Children’s Hospital, University of California, San Diego, San Diego, CA USA; 5 Department of Orthopedic Surgery, National University Hospital (Singapore), 5 Lower Kent Ridge Rd, Singapore, 119074 Singapore; 6 Institut Kassab D’orthopédie, Ksar Said La Manouba, Tunis, Tunisia; 7 Department of Orthopedic Surgery, Columbia University Medical Center, The Och Spine Hospital at New York Presbyterian, New York, NY USA

**Keywords:** Musculoskeletal system, Public health

## Abstract

The evolution of upright bipedalism required coordinated modifications in spinal curvature, pelvic orientation, and lower limb structure. However, it remains unclear whether sagittal alignment traits in modern humans have reached evolutionary stabilization or continue to exhibit developmental variability across populations. We hypothesize that certain sagittal alignment traits have undergone canalization—an evolutionary process that buffers against phenotypic variation—resulting in normal (Gaussian) distributions across populations. Conversely, traits under ongoing biomechanical or developmental constraints may deviate from normality. This study aimed to determine the distribution characteristics of key spinal and pelvic alignment parameters in healthy adults, and to assess whether these distributions reflect evolutionary stabilization or variability. Using high-resolution EOS imaging, we measured ten sagittal alignment parameters in 261 healthy adults under 40 years old across five countries. We applied the Shapiro–Wilk test to assess normality, and evaluated skewness, kurtosis, and modality to characterize distributional shapes. In the full cohort, three parameters—pelvic incidence (PI), lower lumbar lordosis (LL4–S), and spinal 3D length (S3DL)—significantly deviated from normality. PI showed near-symmetric but platykurtic distribution and remained non-normal across sex and age subgroups, while displaying normality within each country. LL4–S showed positive skew and elevated kurtosis. Most other parameters showed unimodal, mesokurtic distributions consistent with evolutionary canalization. Our findings support the hypothesis that sagittal alignment traits in modern humans exhibit varying degrees of evolutionary stabilization. Traits such as PI may reflect both canalization and residual variability, highlighting their relevance in biomechanical adaptation and spinal vulnerability.

**Supplementary Information:**

The online version contains supplementary material available at 10.1038/s41598-025-19366-z.

## Introduction

**Fig. 1:**
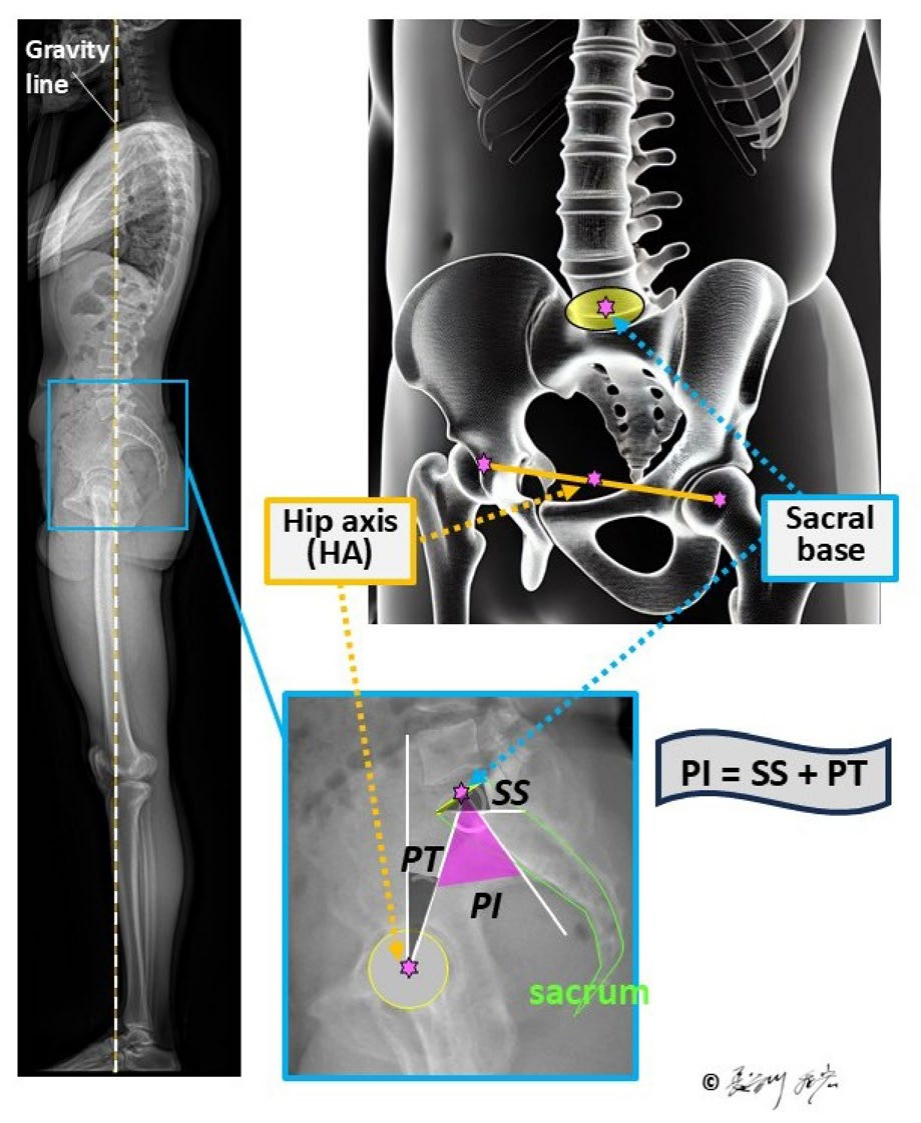
Definition of pelvic incidence (PI) as the sum of sacral slope (SS) and pelvic tilt (PT). Radiographic and anatomical illustrations show the geometric relationships between spine, pelvis, and femoral axis.

 The emergence of bipedal posture marks a pivotal evolutionary adaptation in *Homo sapiens*, driven by evolutionary changes in the entire axial skeleton - from the cranium and foramen magnum position to the shoulder girdle, ribcage, spine, pelvis, and lower limbs. Fossil evidence from early hominins such as *Sahelanthropus tchadensis*,* Orrorin tugenensis*, and *Ardipithecus ramidus* provides insight into this gradual transition. For example, the anteriorly positioned foramen magnum in *S. tchadensis* and femoral morphology in *O. tugenensis* suggest habitual bipedality^1,2^. Similarly, the mosaic pelvic and femoral structures of *A. ramidus* reflect an intermediate stage between arboreal and terrestrial locomotion^3,4^. These anatomical changes facilitated efficient upright locomotion but introduced novel biomechanical demands. Dubousset’s “cone of economy concept” illustrates how minimal muscular effort is needed to maintain balance within a narrow range when spinal and pelvic structures are functionally integrated^5^. The development of lumbar lordosis and pelvic incidence (PI) (Fig. 1) played critical roles in this integration, enabling effective transmission of gravitational and mechanical forces through the axial skeleton^6–9^.

**Fig. 2:**
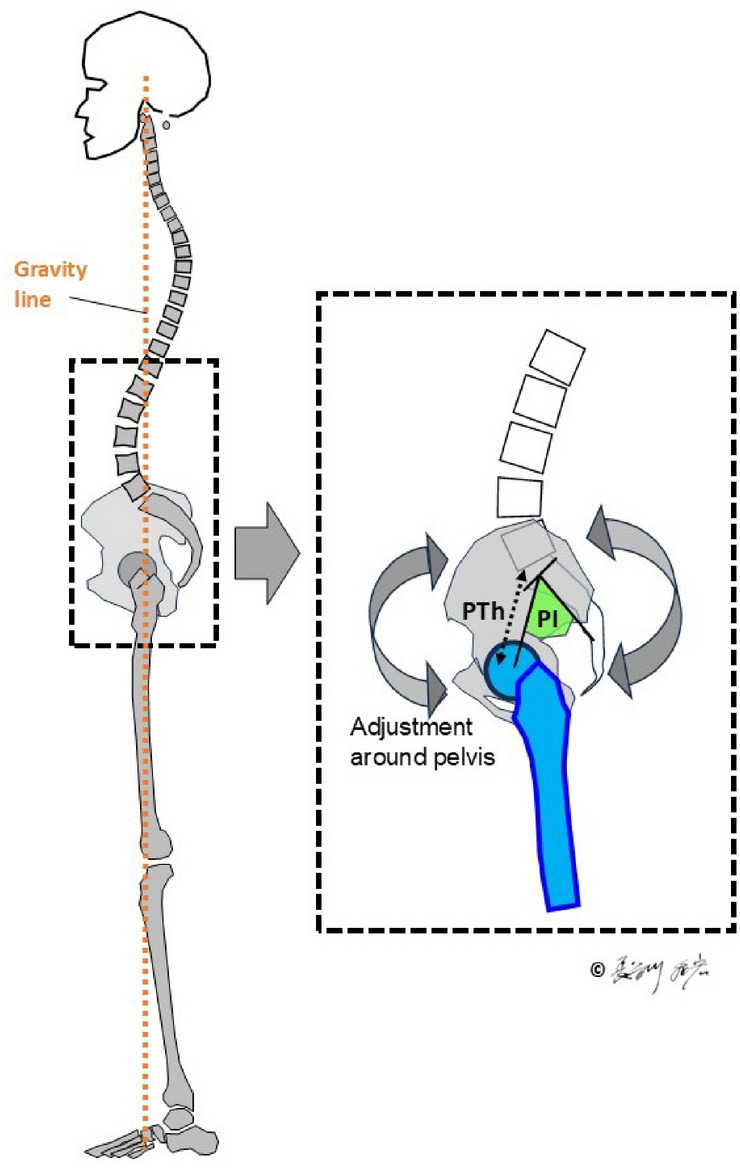
In the standing position, lumbar lordosis and PT are adjusted in relation to the gravity line and PI to maintain postural stability^9^. Pelvic thickness (PTh), representing pelvic size—particularly its cephalocaudal dimension—is also illustrated^11^.

PI is defined as the sum of sacral slope (SS) and pelvic tilt (PT), expressed as PI = SS + PT. SS is the angle formed between the sacral endplate and the horizontal plane, which represents the interaction between the spine and the pelvis, while PT is the angle between the vertical axis and the line connecting the sacral midpoint to the hip axis, highlighting the connection between the pelvis and the lower limb skeleton (Fig. 1)^6,7^. In the evolution of upright posture in hominids, pelvic size, particularly cephalocaudal length, has decreased, while PI and corresponding lumbar lordosis have both increased^8^. In the human standing posture, lumbar lordosis and pelvic retroversion adjust in accordance with PI to maintain sagittal balance (Fig. 2)^9^. Comparative anatomical evidence suggests that the last common ancestor of humans and chimpanzees had a PI ranging between 20° and 40°, comparable to that of extant quadrupedal primates^8,10^. During hominin evolution, the observed inverse relationship between PI and sacro-acetabular distance^8^—later defined as pelvic thickness (PTh)^11^**(Fig. 2**)—indicates positive selection for an increased PI to facilitate upright locomotion. This notion is further reinforced by the strong positive correlation between PI and lumbar lordosis in modern humans (*r* = 0.55, *p* < 0.0001)^12^, highlighting a functional axis that integrates a rigid pelvis with a mobile spine^8,9,13^.

Despite these evolutionary refinements, modern humans remain susceptible to spinal pathologies, including low back pain (LBP)^14,15^, intervertebral disc degeneration^16,17^, and sagittal malalignment^18–20^. These conditions may indicate incomplete evolutionary stabilization or mismatches with modern lifestyles^21^. LBP is one of the most prevalent musculoskeletal conditions worldwide and a major cause of disability. Degenerative disorders affecting the lower lumbar spine and lumbopelvic junction are particularly common, yet the evolutionary or biomechanical reasons for their vulnerability remain unclear. As spine surgeons who regularly treat such conditions, we are driven to understand whether the anatomic and functional properties of this region—especially the lower lumbar spine and pelvis^8^—are evolutionarily optimized or remain morphologically unstable. Given the vulnerability of the human lower back, we focused on two key sagittal alignment parameters: PI and lower lumbar lordosis (LL4–S), which we considered to be biomechanically and evolutionarily central to upright posture.

Previous studies have shown that some skeletal traits follow Gaussian distributions^22,23^, while others deviate, suggesting functional diversity or ongoing adaptation^24,25^. In the principles of stabilizing selection—a well-established evolutionary mechanism—natural selection favors intermediate trait values and eliminates extreme variants. As described by Endler, stabilizing selection typically reduces phenotypic variance and produces tight, symmetric, bell-shaped distributions^26^. Kingsolver & Pfennig similarly note that this form of selection promotes intermediate phenotypes, resulting in unimodal and approximately normal distributions^27^. Hansen also emphasizes that stabilizing selection leads to distributions clustered near an optimal value^28^. This idea aligns with the concept of *canalization*, first introduced by Waddington, which refers to the buffering of developmental pathways against genetic and environmental perturbations^29^. Canalized traits tend to produce consistent phenotypes despite external variation, and their population-level expression often appears as normal distributions, reflecting evolutionary stabilization. Conversely, traits with skewed, heavy-tailed, or multimodal distributions may reflect (1) population heterogeneity (e.g., age, sex or ethnicity), (2) developmental or environmental plasticity, or (3) incomplete or ongoing evolutionary adaptation. This framework builds on Darwinian principles of natural selection, which favor stabilization of functionally critical traits while allowing variation in traits still under evolutionary pressure^30,31^. Here we explored the hypothesis—grounded in evolutionary theory—that traits exhibiting evolutionary stabilization (e.g., through canalization and stabilizing selection) may tend toward normal distributions, while traits under ongoing adaptive pressures could deviate from normality. This framework is interpretive, aiming to generate insights rather than definitive conclusions.

No prior research has systematically evaluated the normality of spinal sagittal alignment parameters across globally diverse populations. To explore the hypothesis, we examined the statistical distribution patterns (normality, skewness, kurtosis, and multimodality) of alignment parameters across healthy individuals from five country populations, leveraging the Multi-Ethnic Alignment Normative Study (MEANS) dataset^32,33^. We analyzed whole-body sagittal alignment using EOS imaging in healthy adults under 40 years of MEANS dataset. We reasoned that if these traits have undergone evolutionary stabilization, their distributions would exhibit symmetry and unimodality—features typically associated with stabilizing selection, with implications for both evolutionary biology and clinical spinal care.

## Materials and methods

### Study design

This study is a large-scale, multi-ethnic, multicenter, cross-sectional radiographic investigation conducted across five countries: Japan, France, Singapore, the United States, and Tunisia. It is part of MEANS^32,33^. All methods were performed in accordance with the relevant guidelines and regulations. Ethical approval was obtained from the institutional review boards (IRB) of all participating centers. In the first author’s IRB, the ethics committee of Medical Corporation Aijinkai approved the study with the committee’s reference number: IRB Approval #6 [R3] – 2021, on September 29, 2021.

### Subjects

Healthy adult volunteers aged 18–80 years were prospectively enrolled at each center to establish normative values for standing sagittal spinal and pelvic alignment. Written informed consent including publication of identifying information/images in an online open-access publication was obtained from all subjects. The following exclusion criteria were applied:

1. Age > 40 years.
2. History of spinal disease or prior spinal surgery.
3. Inability to stand or non-ambulatory status.
4. Oswestry Disability Index (ODI) > 20%^34^.
5. Coronal plane Cobb angle > 20°.
6. Abnormal vertebral counts or transitional vertebrae.

Although 18 years is a common legal and social threshold for adulthood, full skeletal maturity is typically reached by age 25; therefore, individuals aged 18–25 were considered young adults who may still be undergoing residual musculoskeletal maturation.

Given that the prevalence of spondylotic changes increases with age, even among asymptomatic individuals, and that such changes affect spinal alignment^15–17,35–39^, only participants aged 40 years or younger were included in this analysis.

### Demographics measurements

Collected demographic variables included age, sex, body mass index (BMI; weight in kilograms divided by height in meters squared), nationality, and health-related quality of life, assessed via the Oswestry disability index (ODI)^34^. The ODI ranges from 0% (no disability) to 100% (maximum disability).

### Demographics and ODI

The study included 261 healthy participants (149 females, 112 males) aged 18–40 years. The mean age was 29.4 years (SD = 5.9), and the mean BMI was 23.2 kg/m² (SD = 4.5; range: 14.5–40.8). The mean ODI score was 1.8 (SD = 3.7; range: 0–20), indicating minimal disability and confirming the health status of the cohort (Table 1).

**Table 1:** Demographics and Oswestry disability index (ODI).

Parameter	Unit	Mean	Standard Deviation	Min	Max	Confidence intervals	
						Lower 95%	Upper 95%
**Age**	Years old	29.4	5.9	18	40	28.7	30.1
**BMI**	kg/m²	23.2	4.5	14.5	40.8	22.5	23.8
**ODI**	%	1.8	3.7	0	20	1.3	2.2

**BMI**: Body mass index, calculated as weight in kilograms divided by the square of height in meters (kg/m²). **ODI**: Oswestry Disability Index^34^.

### Age and sex distribution by country

Table 2 Presents the age and sex distribution of participants across five countries. Japan contributed the largest sample (*n* = 68; 45 females, 23 males), while Tunisia contributed the smallest (*n* = 30; 19 females, 11 males). Age distribution was comparable across sites, with France having the youngest mean age (26.8 years, SD = 5.2), and Japan the oldest (31.4 years, SD = 5.3).

**Table 2:** Age (years old) and sex divided by five countries.

	Japan	France	Singapore	USA	Tunisia
**Number (F/M)**	68 (45/23)	67 (33/34)	63 (29/34)	33 (23/10)	30 (19/11)
**Age (SD)**	31.4 (5.3)	26.8 (5.2)	29.2 (6.5)	30.1 (4.9)	30.2 (6.0)
**Minimum age**	20	18	20	22	20
**Maximum age**	40	40	40	39	40

**F**: female, **M**: male SD: standard deviation.

### EOS imaging and measurement of spinal alignment

All participants underwent EOS imaging (EOS Imaging, Paris, France), a low-dose, biplanar slot-scanning X-ray system that allows 3D reconstruction and precise measurements of whole axial skeleton in the standing position^40–46^. Participants were scanned in a standardized upright position with their fingertips lightly placed on their cheeks to minimize interference from the upper limbs in sagittal alignment analysis, and gaze fixed via a mirror at eye level.

**Fig. 3:**
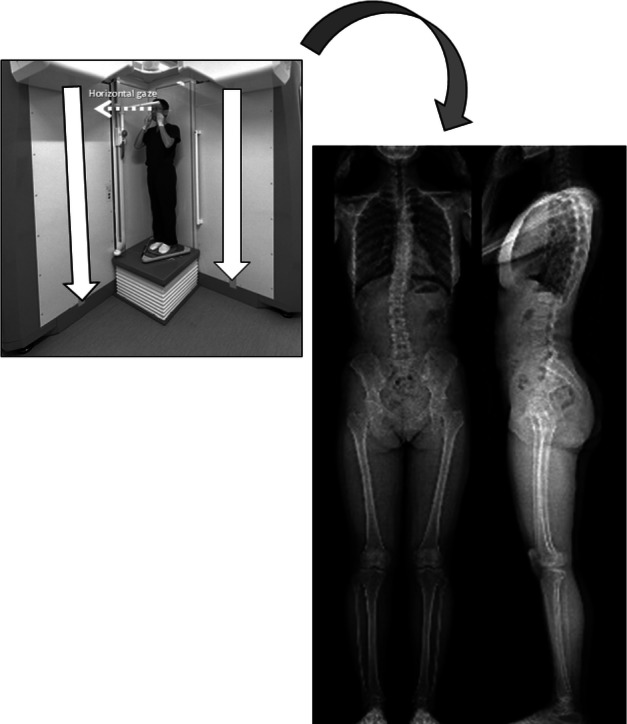
EOS system (a slot-scanning three-dimensional X-ray imager). From the simultaneous anteroposterior and lateral X-rays of the whole body to the 3D bone external envelope technique, 3D reconstruction is possible at every level of the osteoarticular system and especially the spine in the standing position. The subject is the author (K.H.).

**Fig. 4:**
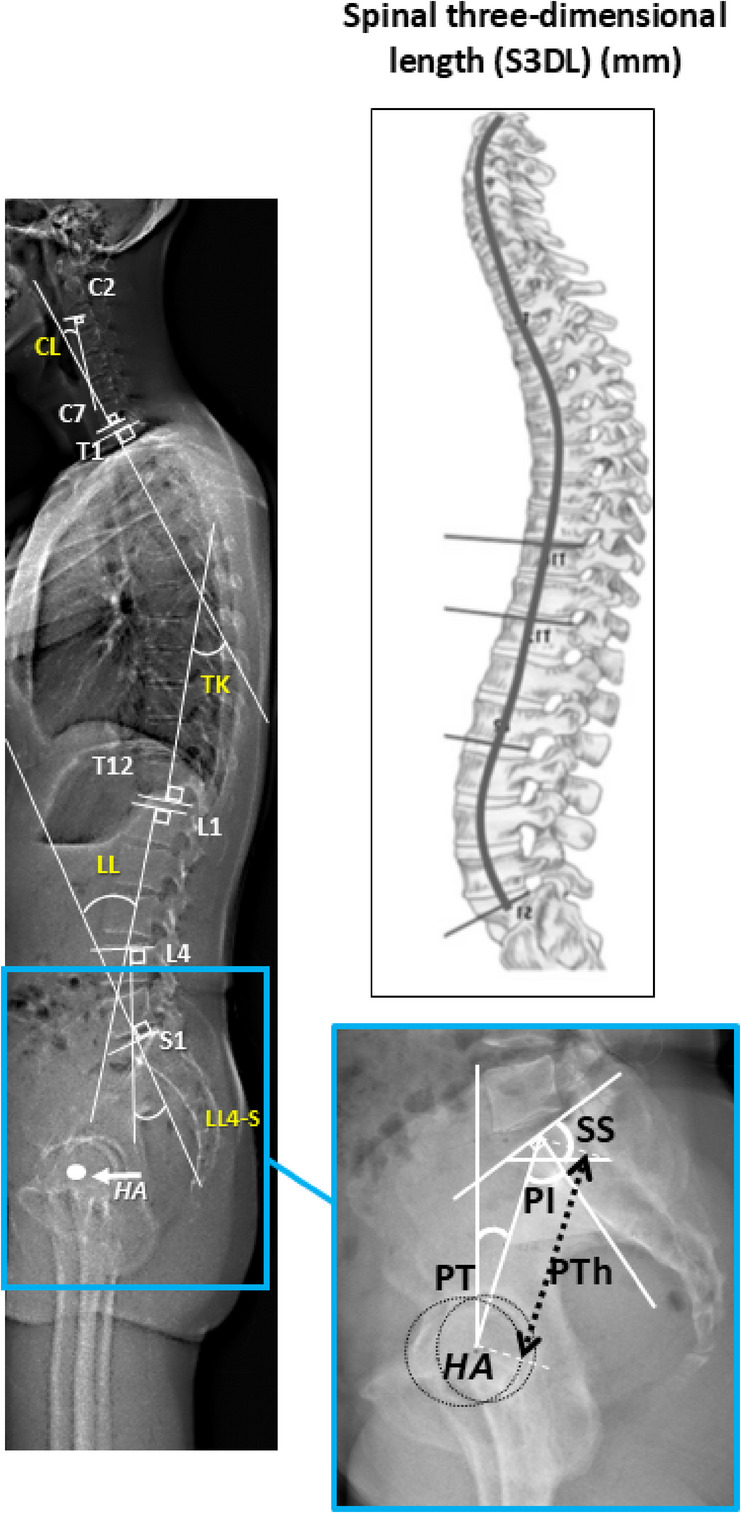
Key sagittal alignment parameters measured on EOS^9^.

The EOS scan speed was set at 7.6 cm/s, with acquisition time adjusted to height (Fig. 3)**.** Sagittal alignment parameters measured included (Fig. 4)^9^:

1. Cervical lordosis (CL; C2–C7).

The angle between the inferior endplate of C2 and the inferior endplate of C7, reflecting the curvature of the cervical spine.

(2) Thoracic kyphosis (TK; T1–T12).

The angle between the superior endplate of T1 and the inferior endplate of T12, representing the thoracic spine curvature.

(3) Lumbar lordosis (LL; L1–S1).

The angle between the superior endplate of L1 and the superior endplate of S1, indicating overall lumbar curvature.

(4) Upper lumbar lordosis (LL1–4).

The angle between the superior endplate of L1 and the inferior endplate of L4, reflecting lordosis in the upper lumbar region.

(5) Lower lumbar lordosis (LL4–S).

The angle between the superior endplate of L4 and the superior endplate of S1, representing lower lumbar curvature.

(6) Sacral slope (SS).

The angle between the superior endplate of S1 and a horizontal reference line.

(7) Pelvic tilt (PT).

The angle between the line connecting the midpoint of the sacral plate to the femoral heads axis and the vertical reference line.

(8) Pelvic incidence (PI).

PI was measured as the angle between the perpendicular to the sacral plate at its midpoint and the line connecting this point to the femoral head axis (Fig. 1)^6,7^.

(9) Pelvic thickness (PTh)^11^.

PTh represents pelvic size, particularly cephalocaudal length (Fig. 2).

(10) The spinal three-dimensional length (S3DL).

S3DL was defined as the 3D curvilinear path from the odontoid tip to the center of S1 endplate all through the vertebral centers^46^(Fig. 4).

### Statistical analysis

Continuous demographic variables (age, BMI, ODI) were summarized using means, standard deviations (SD), ranges, and 95% confidence intervals. Alignment parameters were assessed for univariate distribution by calculating mean, SD, and quartiles (Q1–Q4). Normality of each parameter was tested using the Shapiro–Wilk test. A p-value < 0.05 was considered indicative of non-normal distribution. Supplementary assessments included boxplots and quantile–quantile (Q–Q) plots.

In addition to the Shapiro–Wilk test, we evaluated distributional characteristics, whether deviations arise from asymmetry, heavy tails, or both, using skewness (D’Agostino test), kurtosis (Anscombe–Glynn test), and multimodality (number of peaks) to better understand the nature of deviation from normality. To evaluate multimodality, we applied Hartigan’s dip test, which quantifies the maximum difference between the empirical distribution function and the best-fitting unimodal distribution, providing a sensitive measure of multimodal structure. A p-value < 0.05 was considered indicative of significant deviation from unimodality.

- **Skewness** quantifies asymmetry; a skewness of 0 indicates a perfect symmetrical distribution. Near-zero skewness suggests trait symmetry around an optimal mean, while positive or negative skew may reflect directional selection or population heterogeneity.
- **Kurtosis** measures the peakedness or tailedness of a distribution. A kurtosis of 3 represents a normal (mesokurtic) distribution. Higher values (> 3, leptokurtic) suggest more peaked distributions with heavier tails, while lower values (< 3, platykurtic) indicate flatter, broader distributions.
- **Multimodality** describes the number of distinct peaks. A unimodal distribution suggests stabilizing selection toward a common phenotype. Multimodal patterns (≥ 2 peaks) may indicate subpopulations, alternative morphologies, or transitional evolutionary states.

Normality tests for spinal alignment parameters were also conducted within subgroups defined by country (five countries), sex (female/male), and age (< 30 years and ≥ 30 years). To examine the potential influence of age on the distribution of spinal alignment parameters, we divided the sample into two age groups. Given the total sample size (*n* = 261), further subdivision into multiple smaller age groups was considered inappropriate, as it could compromise the reliability of the normality tests. Therefore, based on the observed mean age of 29.0 years and median age of 29.4 years, we selected 30 years as a natural cutoff and categorized participants into two groups accordingly.

All statistical analyses were performed using JMP software (version 9; SAS Institute, Cary, NC, USA). A significance threshold of *p* < 0.05 was applied throughout.

## Results

### Sagittal alignment parameters by country (Supplementary Table 1)

Analysis of variance (ANOVA) across five countries (Japan, France, Singapore, USA, and Tunisia) revealed significant inter-country differences in five of the ten alignment parameters. TK (*p* = 0.0018), LL (*p* < 0.0001), LL4–S (*p* < 0.0001), SS (*p* = 0.0203), and S3DL (*p* = 0.0214) all showed statistically significant differences. The post-hoc power analysis confirmed adequate statistical power (≥ 0.78) for these parameters. In contrast, parameters such as CL, LL1–4, PT, PI, and PTh did not differ significantly among the five countries (*p* > 0.05), and their associated power values were lower, suggesting possible sample size limitations for detecting subtle differences.

**Fig. 5:**
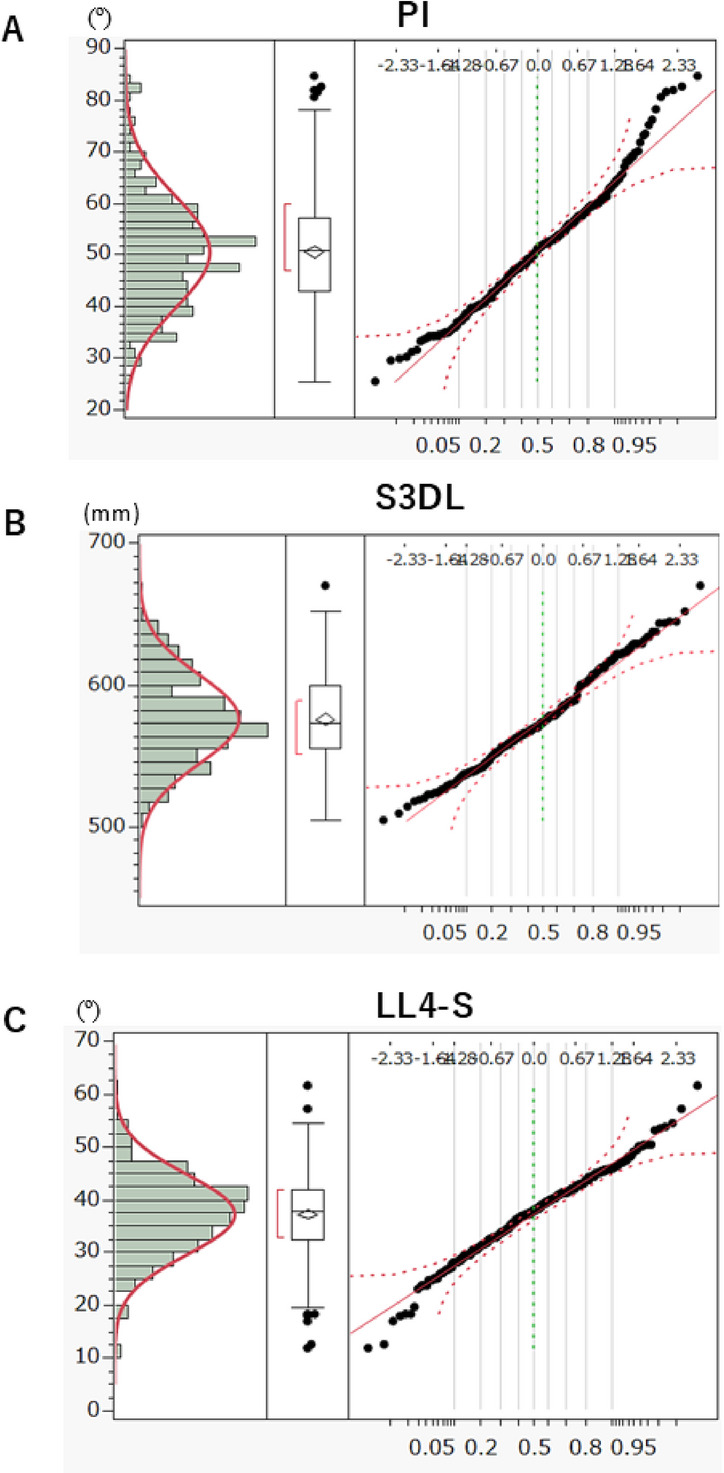
Normality fitting for continuous univariate distribution with box plot of outliers and the normal Q-Q Plot (Quantile-Quantile Plot) in full population (*n*=261). **A**: Pelvic incidence (PI), **B**: Spinal three-dimensional length (S3DL), **C**: Lumbar lordosis of L4-S1 (LL4-S).

### Sagittal alignment parameters and normality test: full population (*n* = 261) (Table 3, Fig. 5)

Table 3 summarizes sagittal alignment values and results of the Shapiro–Wilk test for normality. Most parameters—CL, TK, LL, LL1–4, SS, PT, and PTh—exhibited normal distributions. However, three parameters, PI (W = 0.9805, *p* = 0.0012), S3DL (W = 0.9885, *p* = 0.0365), and LL4–S (W = 0.9891, *p* = 0.0474), deviated significantly from normality.

Despite the mean PI (50.7°) equaling the median, the distribution was spindle-shaped and exhibited the most significant deviation from normality.

**Table 3:** Sagittal alignment values and normality fitting test by Shapiro-Wilk W test (*n*=261).

Parameters	Mean *(*SD)	1^st^ quartile	2^nd^ quartile	3^rd^ quartile	4^th^ quartile	Shapiro-Wilk W test	
						W value	*p* value
**CL** (°)	- 2.2 (12.4)	- 45.7 ~ - 10.8	- 10.8 ~ - 1.3	- 1.3 ~ 6.6	6.6 ~ 24.7	0.992162	0.1819
**TK** (°)	41.9 (10.2)	14.4 ~ 34.2	34.2 ~ 42.6	42.6 ~ 48.4	48.4 ~ 73.1	0.995795	0.7066
**LL** (°)	57.4 (11.0)	24.4 ~ 49.7	49.7 ~ 57.5	57.5 ~ 64.0	64.0 ~ 86.1	0.995754	0.6987
**LL1-4** (°)	20.1 (8.1)	- 5.4 ~ 15.0	15.0 ~ 20.2	20.2 ~ 25.7	25.7 ~ 42.5	0.996929	0.8990
**LL4-S (°)**	**37.3 (7.6)**	**11.8 ~ 32.6**	**32.6 ~ 37.8**	**37.8 ~ 42.0**	**42.0 ~ 61.4**	**0.989129**	**0.0474**
**SS** (°)	39.8 (8.1)	20.2 ~ 34.2	34.2 ~ 39.8	39.8 ~ 44.7	44.7 ~ 65.6	0.994021	0.3908
**PT** (°)	10.9 (6.6)	- 6.9 ~ 6.8	6.8 ~ 10.7	10.7 ~ 15.2	15.2 ~ 31.5	0.993897	0.3726
**PI** (°)	**50.7 (10.6)**	**25.5 ~ 42.9**	**42.9 ~ 50.7**	**50.7 ~ 57.1**	**57.1 ~ 84.5**	**0.980460**	**0.0012**
**PTh** (mm)	106.9 (7.4)	88.1 ~ 102.4	102.4 ~ 106.9	106.9 ~ 111.7	111.7 ~ 129.2	0.995157	0.5880
**S3DL** (mm)	**576.1 (31.1)**	**504.4 ~ 555.4**	**555.4 ~ 572.8**	**572.8 ~ 599.7**	**599.7 ~ 670.1**	**0.988544**	**0.0365**

**SD**: standard deviation. **CL**: Cervical lordosis, **TK**: Thoracic kyphosis, **LL**: Lumbar lordosis of L1 to S1, **LL1-4**: Lumbar lordosis of L1 to L4, **LL4-S**: Lumbar lordosis of L4-S1, **SS**: Sacral slope, **PT**: Pelvic tilt, **PI**: Pelvic incidence, **PTh**: Pelvic thickness, **S3D**L: Spinal three-dimensional length. The Shapiro-Wilk W test with a p-value < 0.05 indicates that the data deviates from a normal distribution (**bold character**).

### Normality test of sagittal alignment parameters by country (Supplementary Table 2)

The Shapiro–Wilk normality test results for 10 alignment parameters across five countries revealed that the majority of parameters exhibited no significant deviation from normality within each population (*p* > 0.05). However, two parameters—LL4–S in Singapore (*p* = 0.0272) and S3DL in the USA (*p* = 0.0015)—showed statistically significant deviations from a normal distribution.

Importantly, PI, which showed non-normality in the full dataset, did not exhibit statistically significant deviation from normality in any of the five countries individually (*p* > 0.05).

### Distribution characteristics of alignment parameters by skewness, kurtosis, and multimodality analysis (Table 4)

Among the ten alignment parameters evaluated, two—LL4–S and PI—demonstrated significant deviations from a normal distribution based on skewness and/or kurtosis analysis:

- PI exhibited a low skewness (skewness = 0.494, *p* = 0.0015) and mesokurtic kurtosis (3.489, *p* = 0.113), indicating that the deviation from normality was primarily driven by subtle asymmetry rather than peakedness or heavy tails.
- LL4–S exhibited significant negative skewness (skewness = − 0.299, *p* = 0.0468) and elevated kurtosis (kurtosis = 3.74, *p* = 0.0337), suggesting a left-skewed distribution with heavy tails.
- CL was the only parameter to exhibit a multimodal distribution (two peaks), despite showing no significant skewness or kurtosis. This bimodal pattern may reflect the presence of distinct subgroups within the population—such as individuals with straighter cervical profiles versus those with pronounced lordosis—potentially due to anatomical variation, habitual posture, or measurement variability.

All remaining parameters—including TK, LL, LL1–4, SS, PT, PTh, and S3DL—demonstrated approximate normality, with unimodal distributions and non-significant values for both skewness and kurtosis.

**Fig. 6:**
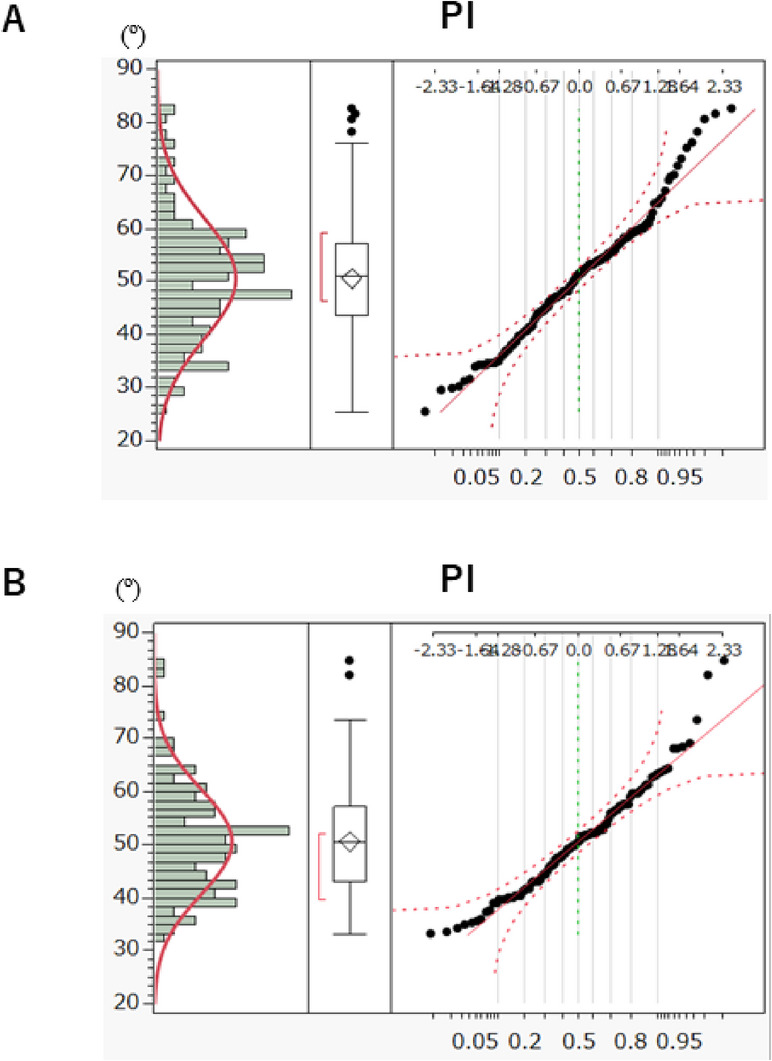
Normality fitting for continuous univariate distribution with box plot of outliers and the normal Q-Q Plot (Quantile-Quantile Plot) of Pelvic incidence (PI) in the female group, *n*=149, (**A**) and the male group, *n*=112, (**B**).

**Table 4:** Distribution characteristics of alignment Parameters.

Parameter	Skewness (*p*-value)	Kurtosis (*p*-value)	Multimodality (number of peaks)
**CL**	−0.15783 (0.28778)	2.79255 (0.56813)	2
**TK**	0.12972 (0.38146)	2.87091 (0.79851)	1
**LL**	−0.01214 (0.93450)	3.09468 (0.60058)	1
**LL1-4**	−0.16081 (0.27888)	3.09468 (0.50476)	1
**LL4-S**	−0.29938 (**0.04680**)	3.74085 (**0.03370**)	1
**SS**	0.240611 (0.10767)	3.11197 (0.56364)	1
**PT**	0.095492 (0.51865)	3.33508 (0.22689)	1
**PI**	0.493997 (**0.00146**)	3.48867 (0.11303)	1
**PTh**	0.033559 (0.82023)	3.13893 (0.50933)	1
**S3DL**	0.264573 (0.07768)	2.62229 (0.17454)	1

Skewness, kurtosis, and multimodality (number of peaks) were tested by using D’Agostino test, Anscombe–Glynn test, and Hartigan’s dip test, respectively. In multimodality test, a p-value < 0.05 was considered indicative of significant deviation from unimodality.

### Normality test of sagittal alignment parameters by age group (Supplementary Table 3)

The Shapiro–Wilk normality test was conducted separately for younger (< 30 years) and older *(>* 30 years) age groups across ten sagittal alignment parameters. In the < 30 group, four parameters—SS, PI, PTh, and S3DL—showed significant deviation from normality (*p* < 0.05), with the strongest non-normality observed in S3DL (*p* = 0.0032) and PI (*p* = 0.0049). In contrast, the *≥* 30 group exhibited overall improved normality, with no parameters reaching statistical significance for non-normality.

### Normality test of sagittal alignment parameters by sex (Tables 5 and 6)

#### Female subgroup (*n* = 149)

In females, only PI deviated from normality (W = 0.9806, *p* = 0.0337). All other parameters—including S3DL and LL4–S—followed normal distributions (Table 5, Fig. 6A). The mean S3DL in females was 558.6 mm (SD = 21.2), slightly shorter than the overall average.

**Table 5:** Values of alignment parameters in sagittal plane X-ray measurement and normality fitting test by Shapiro-Wilk W test in the female group (*n*=149).

Parameters	Mean *(*SD)	1^st^ quartile	2^nd^ quartile	3^rd^ quartile	4^th^ quartile	Shapiro-Wilk W test	
						W value	*p* value
**CL** (°)	- 4.5 (12.9)	- 45.7 ~ - −13.9	- 13.9 ~ - −5.5	- 5.5 ~ 4.5	4.5 ~ 23.2	0.991177	0.4815
**TK** (°)	41.0 (10.4)	16.5 ~ 33.4	33.4 ~ 41.8	41.8 ~ 47.4	47.4 ~ 73.1	0.988754	0.2754
**LL** (°)	58.5 (10.7)	28.1 ~ 50.4	50.4 ~ 59.1	59.1 ~ 65.8	65.8 ~ 86.1	0.995660	0.9408
**LL1-4** (°)	21.2 (8.1)	2.0 ~ 15.9	15.9 ~ 21.1	21.0 ~ 26.5	26.5 ~ 42.5	0.994988	0.8916
**LL4-S** (°)	37.3 (7.9)	11.8 ~ 32.0	32.0 ~ 37.8	37.8 ~ 42.0	42.0 ~ 61.4	0.993212	0.7081
**SS** (°)	40.1 (8.1)	20.2 ~ 34.5	34.5 ~ 39.6	39.6 ~ 44.9	44.9 ~ 65.6	0.989315	0.3155
**PT** (°)	10.6 (7.2)	- 6.0 ~ 6.2	6.2 ~ 10.4	10.4 ~ 15.2	15.2 ~ 31.5	0.991180	0.4818
**PI** (°)	**50.7 (11.2)**	**25.5 ~ 43.5**	**43.5 ~ 51.0**	**51.0 ~ 57.1**	**57.1 ~ 82.6**	**0.980626**	**0.0337**
**PTh** (mm)	106.6 (7.1)	88.3 ~ 103.3	103.3 ~ 107.7	107.7 ~ 112.0	112.0 ~ 128.7	0.990922	0.4557
**S3DL** (mm)	558.6 (21.2)	504.4 ~ 541.5	541.5 ~ 560.4	560.4 ~ 573.6	573.6 ~ 612.1	0.990945	0.4580

The Shapiro-Wilk W test with a p-value < 0.05 indicates that the data deviates from a normal distribution (**bold character**).

#### Male subgroup (*n* = 112)

In males, both PI (W = 0.9674, *p* = 0.0077) and LL1–4 (W = 0.9750, *p* = 0.0338) deviated from normality, while S3DL was normally distributed (Table 6, Fig. 6B). The mean S3DL in males was 599.4 mm (SD = 26.7), significantly longer than in females, consistent with known sex-related differences in spinal morphology. 

**Table 6:** Values of alignment parameters in sagittal plane X-ray measurement and normality fitting test by Shapiro-Wilk W test in the male group (n=112).

Parameters	Mean *(*SD)	1^st^ quartile	2^nd^ quartile	3^rd^ quartile	4^th^ quartile	Shapiro-Wilk W test	
						W value	*p* value
**CL** (°)	0.8 (11.0)	- 24.6 ~ - −7.0	- 7.0 ~ 1.2	1.2 ~ 8.6	8.6 ~ 24.7	0.990735	0.6503
**TK** (°)	43.0 (9.8)	14.4 ~ 37.1	37.1 ~ 43.6	43.6 ~ 49.5	49.5 ~ 63.9	0.992303	0.7881
**LL** (°)	56.0 (11.3)	24.4 ~ 49.0	49.0 ~ 56.6	56.6 ~ 63.1	63.1 ~ 84.5	0.989955	0.5815
**LL1-4** (°)	18.8 (8.0)	- 5.4 ~ 13.7	13.7 ~ 19.4	19.4 ~ 23.9	23.9 ~ 34.2	0.982109	0.1395
**LL4-S** (°)	**37.3 (7.1)**	**12.4 ~ 32.9**	**32.9 ~ 37.5**	**37.5 ~ 42.4**	**42.4 ~ 54.3**	**0.975027**	**0.0338**
**SS** (°)	39.4 (8.2)	20.4 ~ 33.6	33.6 ~ 40.0	40.0 ~ 44.2	44.2 ~ 61.7	0.994022	0.9137
**PT** (°)	11.2 (5.7)	- 6.9 ~ 8.3	8.3 ~ 11.4	11.4 ~ 15.1	15.1 ~ 25.8	0.991726	0.7385
**PI** (°)	**50.6 (9.9)**	**33.1 ~ 42.9**	**42.9 ~ 50.3**	**50.3 ~ 57.1**	**57.1 ~ 84.5**	**0.967356**	**0.0077**
**PTh** (mm)	106.0 (7.8)	88.1 ~ 100.7	100.7 ~ 105.9	105.9 ~ 111.0	111.0 ~ 129.2	0.994363	0.9325
**S3DL** (mm)	599.4 (26.7)	524.3 ~ 578.2	578.2 ~ 602.0	602.0 ~ 619.7	619.7 ~ 670.1	0.990067	0.5913

The Shapiro-Wilk W test with a p-value < 0.05 indicates that the data deviates from a normal distribution (**bold character**).

## Discussion

**Fig. 7:**
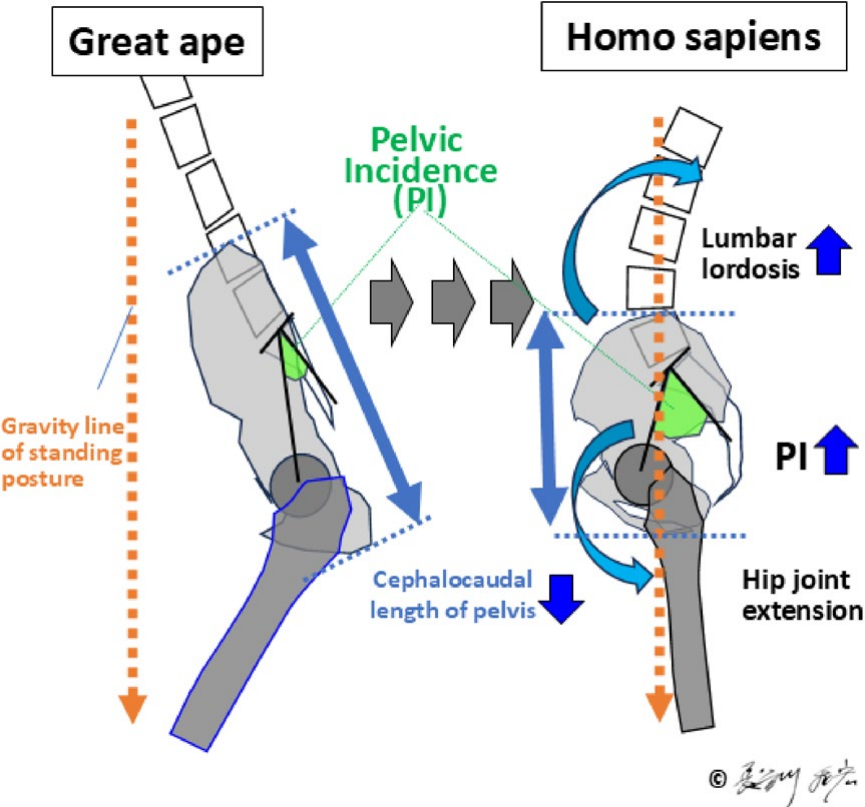
Schema of evolution of lumbar spine, pelvis, and hip joints for bipedal standing.

The evolutionary origins of the modern human skeleton can be traced back to the emergence of habitual bipedal posture, which began approximately 7 million years ago. Fossil evidence from early hominins such as *S. tchadensis*^1^, *O. tugenensis*^2^, and *A. ramidus*^3^ highlights a gradual shift from arboreal to terrestrial locomotion. The pelvic and femoral structures of *A. ramidus* illustrate a mosaic of adaptations for upright walking^4^, suggesting that these traits may reflect ongoing variation shaped by biomechanical and evolutionary pressures during a transitional phase of adaptation.

**Fig. 8:**
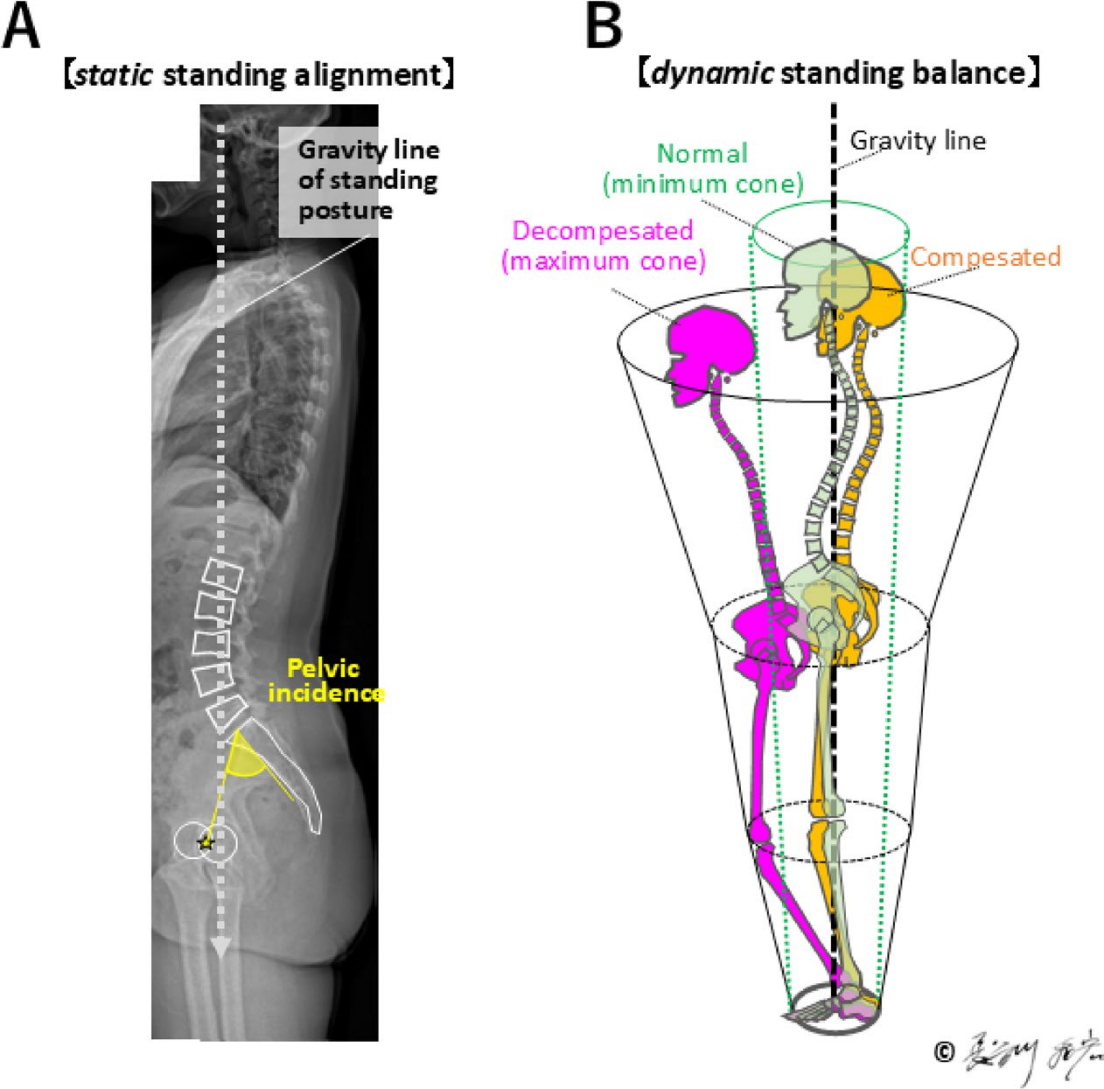
Schema of *static* standing alignment of human in the sagittal plane with gravity line (**A**) and *dynamic* standing balance (**B**).

The maintenance of upright posture and gait is supported by the evolution of lumbar lordosis and pelvic morphology, particularly by an increase in PI^8,9^ (Fig. 7). This shift facilitated efficient load transmission along the spine and lower extremities, optimizing bipedal locomotion (Fig. 8**A**). In the upright stance, the human axial skeleton is vertically aligned, enabling balance and energy efficiency. Dubousset’s concept of the “cone of economy” illustrates how a stable postural alignment permits minimal muscular effort within a narrow range around the gravity line, while impaired alignment expands this cone and demands greater muscular compensation (Fig. 8**B**)^5^.

The lower lumbar spine and pelvis constitute the core of sagittal alignment in upright posture, playing a crucial role in maintaining bipedal balance and energy-efficient standing. However, from a clinical perspective, these same regions are disproportionately affected by degenerative changes^16–20,35–39^and are leading sources of mechanical low back pain worldwide^14,15^. This paradox raises the question of whether the morphology of this region is truly optimized or remains evolutionarily and functionally vulnerable.

In this study, we analyzed ten key sagittal alignment parameters in a healthy young population across five countries using EOS imaging, a low-dose, high-resolution full-body X-ray modality. By focusing on a cohort *≤* 40 years old, we aimed to minimize the confounding effects of age-related degenerative changes and better capture innate structural variability.

We hypothesized that traits under strong evolutionary stabilization would show normal distributions due to canalization—a developmental buffering process that reduces phenotypic variation—whereas traits with ongoing adaptive or developmental variability might deviate from normality^26–29^. While this framework draws on evolutionary theory, our interpretation remains exploratory and does not claim direct causal inference. Rather, we propose that the presence or absence of distributional normality may serve as a signal for further inquiry into the developmental and biomechanical significance of sagittal alignment traits.

Although PI followed normal distributions within each individual country, it consistently deviated from normality in the global cohort and across sex and age subgroups. This pattern suggests underlying structural or developmental variability not solely attributable to regional or ethnic differences. Rather than implying ongoing evolutionary change, the observed distribution may reflect residual adaptive variation or population-level morphological diversity shaped by biomechanical demands (Tables 3, 4, Supplementary Table 2).

In the global analysis, three parameters - PI, S3DL, and LL4–S - deviated significantly from normality. PI demonstrated moderate skewness (0.49) and near-mesokurtic distribution (kurtosis 3.49), implying a unimodal but broad distribution. This may reflect evolutionary canalization, where stabilizing selection maintains a core functional range while permitting peripheral diversity suited to different morphotypes. In contrast, LL4–S exhibited slightly lower skewness (− 0.30) and slightly higher kurtosis (3.74), pointing to asymmetric variation biased toward lower lordosis values, potentially reflecting anatomical vulnerability in this biomechanically sensitive segment (Table 4).

**Fig. 9:**
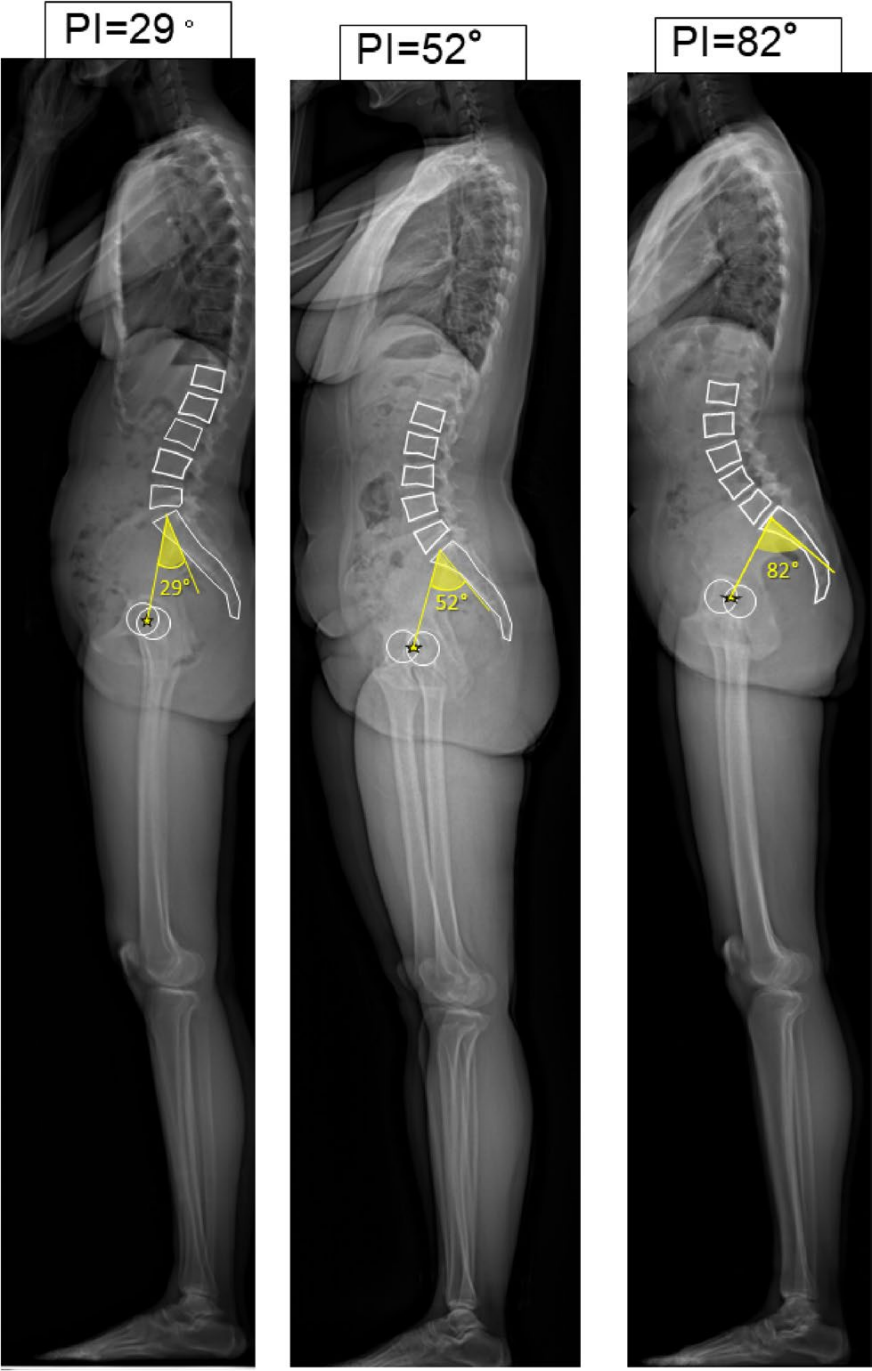
Whole body sagittal alignment of the subjects with low PI (29°), normal PI (52°), and high PI (82°). Lumbar vertebrae, L1 to L5, Sacrum, and bilateral femoral heads are delineated with PI values, respectively.

PI quantifies the geometric relationship between the sacrum and femoral heads^6,7^. Its evolutionary increase has been instrumental in enabling upright posture and lumbar lordosis^8,9,13^. However, in modern humans, PI spans a wide range—from 20° to over 80°—and emerged as the most statistically non-normal parameter in our dataset (Table 3, Fig. 9). This variability prompts key questions: Has PI not reached an evolutionarily stable state? Or does its diversity represent a strategy for biomechanical adaptability? PI likely embodies both evolutionary innovation and mechanical trade-offs: high PI is associated with spondylolisthesis, while low PI is linked to flat-back syndrome and discogenic disease^47,48^. These associations suggest that variation in PI may underlie differing patterns of spinal degeneration, particularly with aging^14,15,39^.

Cross-national comparison by ANOVA revealed that while several spinal parameters—especially thoracic and lumbar curvature and spinal length—varied significantly between countries, PI remained consistent across populations (Supplementary Table 1). Furthermore, normality tests showed that PI was not significantly non-normal within any single country (Supplementary Table 2). These findings support the idea that some parameters (e.g., LL4–S, S3DL) reflect population-level variation, whereas PI may be constrained by a globally conserved morphological pattern.

Age subgroup analysis also yielded noteworthy trends. Among individuals < 30 years old, four parameters—SS, PI, PTh, and S3DL—showed significant non-normality, while those > 30 showed no such deviations. This may reflect greater developmental variability or less biomechanical consolidation in early adulthood (Supplementary Table 3). Bailey et al. similarly reported that while lumbar lordosis increases with age, PI remains stable during development, and the correlation between PI and LL emerges only in older age groups^49^. Our findings are consistent with this developmental trajectory.

Sex-specific analyses revealed additional complexity: PI deviated from normality in both males and females, LL4–S was non-normal in males only, and S3DL was non-normal only in the pooled cohort (Tables 5, 6). These findings align with known sexual dimorphism in pelvic and spinal morphology^46^ and underscore the multifactorial influences on sagittal alignment.

### Study limitations

First, environmental factors—such as sedentary lifestyle and nutrition—may influence skeletal morphology independently of evolutionary processes^21^. Second, developmental and genetic constraints could limit trait variability or shape distributions, complicating interpretations of evolutionary stability^50^. Third, this cross-sectional dataset provides only a snapshot of modern humans; longitudinal data would be needed to track ongoing adaptive processes over time. Also when we perform statistical analysis within individual countries, limited sample size or reduced statistical power may influence the results.

## Conclusion

This international study investigated whether sagittal spinal alignment traits in healthy young adults exhibit distributional patterns consistent with evolutionary stabilization. Our hypothesis proposed that canalized traits would follow normal distributions, while traits still shaped by biomechanical or developmental variability might deviate. The findings supported this framework: most alignment parameters, including thoracic kyphosis and lumbar lordosis, showed Gaussian distributions suggestive of canalization, while PI and LL4–S deviated from normality in the global cohort. Notably, PI showed persistent non-normality across sex and age groups despite normality within regional populations, implying residual adaptive variation. These patterns suggest that certain fundamental traits of the human skeleton—particularly those critical to standing posture and balance—may still reflect evolutionary, biomechanical, or environmental pressures. Understanding such evolutionary signals is essential for anticipating future challenges in spinal health and informing preventive strategies in an aging and globally diverse population.

## Supplementary Information

Below is the link to the electronic supplementary material.

Supplementary Material 1

Supplementary Material 2

Supplementary Material 3

## Data Availability

The data that support the findings of this study are not openly available due to reasons of sensitivity and are available from the corresponding author upon reasonable request. Data are located in controlled access data storage in the Springer Nature figshare repository.
