# Supplementary material for: Normality of sagittal spinal alignment parameters reveals evolutionary signals in healthy adults across five countries
Source: Sci Rep. 2025 Oct 10;15:35484. doi: 10.1038/s41598-025-19366-z (PMC12514220; doi:10.1038/s41598-025-19366-z)
Supplement: Supplementary file 1 — Supplementary Material 1 [file 41598_2025_19366_MOESM1_ESM.docx]

**Supplementary Table 1. Alignment difference among five countries by ANOVA**

| **Parameters** | **Japan (n=68)** | **France (n=67)** | **Singapore (n=63)** | **USA (n=33)** | **Tunisia (n=30)** |  |  |  |
| --- | --- | --- | --- | --- | --- | --- | --- | --- |
|  |  |  |  |  |  | **p value** | **Power** | **MSS** |
| **CL** (°) | -5.3 (1.5) | 0.23 (1.5) | -1.1 (1.6) | -1.3 (2.1) | -4.1 (2.2) | 0.0857 | 0.6116 | 303.4 |
| **TK** (°) | 40.2 (1.2) | 43.0 (1.2) | 38.8 (1.3) | 46.0 (1.7) | 45.2 (1.8) | **0.0018** | **0.9346** | **143.8** |
| **LL** (°) | 57.3 (1.3) | 58.1 (1.3) | 51.6 (1.3) | 63.4 (1.8) | 61.9 (1.9) | **<0.0001** | **0.9994** | **71.7** |
| **LL1-4** (°) | 19.4 (1.0) | 19.4 (1.0) | 19.2 (1.0) | 23.2 (1.4) | 22.1 (1.5) | 0.0797 | 0.6227 | 296.8 |
| **LL4-S (°)** | 38.7 (0.9) | 38.7 (0.9) | 32.4 (0.9) | 40.3 (1.2) | 39.7 (1.3) | **<0.0001** | **0.9999** | **61.4** |
| **SS** (°) | 41.0 (1.0) | 39.4 (1.0) | 37.1 (1.0) | 41.9 (1.4) | 41.3 (1.5) | **0.0203** | **0.7885** | **212.7** |
| **PT** (°) | 10.5 (0.8) | 11.0 (0.8) | 11.7 (0.8) | 10.4 (1.2) | 10.2 (1.2) | 0.7775 | 0.1535 | 1401.3 |
| **PI** (°) | 51.5 (1.3) | 50.4 (1.3) | 48.9 (1.3) | 52.3 (1.9) | 51.4 (1.9) | 0.5296 | 0.2532 | 782.9 |
| **PTh** (mm) | 107.0 (0.9) | 107.7 (0.9) | 108.0 (0.9) | 103.9 (1.3) | 105.8 (1.3) | 0.0867 | 0.6099 | 304.5 |
| **S3DL** (mm) | 570.6 (3.7) | 586.8 (3.7) | 571.9 (3.9) | 575.4 (5.3) | 574.1 (5.6) | **0.0214** | **0.7835** | **215.0** |

Data are shown in mean (standard deviation)

A p-value < 0.05 indicates that there significant difference among countries (**bold character**).

**Power**: statistical power

**MSS**: Minimum sample size for statistical significance
