# Supplementary material for: Normality of sagittal spinal alignment parameters reveals evolutionary signals in healthy adults across five countries
Source: Sci Rep. 2025 Oct 10;15:35484. doi: 10.1038/s41598-025-19366-z (PMC12514220; doi:10.1038/s41598-025-19366-z)
Supplement: Supplementary file 2 — Supplementary Material 2 [file 41598_2025_19366_MOESM2_ESM.docx]

**Supplementary Table 2. Normality test of sagittal alignment parameters by country**

|  | **Japan (n=68)** | | **France (n=67)** | | **Singapore (n=63)** | | **USA (n=33)** | | **Tunisia (n=30)** | |
| --- | --- | --- | --- | --- | --- | --- | --- | --- | --- | --- |
| **Parameters** | **Shapiro-Wilk W test** | | **Shapiro-Wilk W test** | | **Shapiro-Wilk W test** | | **Shapiro-Wilk W test** | | **Shapiro-Wilk W test** | |
|  | **W value** | **p value** | **W value** | **p value** | **W value** | **p value** | **W value** | **p value** | **W value** | **p value** |
| **CL** (°) | 0.979976 | 0.3435 | 0.986613 | 0.6904 | 0.978997 | 0.3553 | 0.966315 | 0.3857 | 0.962762 | 0.3637 |
| **TK** (°) | 0.981663 | 0.4161 | 0.986744 | 0.6979 | 0.975049 | 0.2286 | 0.990672 | 0.9913 | 0.956587 | 0.2528 |
| **LL** (°) | 0.981532 | 0.4101 | 0.985430 | 0.6230 | 0.983169 | 0.5429 | 0.961869 | 0.2917 | 0.974592 | 0.6707 |
| **LL1-4** (°) | 0.988609 | 0.7944 | 0.985680 | 0.6371 | 0.990738 | 0.9190 | 0.968101 | 0.4296 | 0.956138 | 0.2460 |
| **LL4-S (°)** | 0.991834 | 0.9388 | 0.984990 | 0.5982 | 0.956928 | **0.0272** | 0.987389 | 0.9602 | 0.984356 | 0.9257 |
| **SS** (°) | 0.9990626 | 0.8934 | 0.992261 | 0.9541 | 0.990842 | 0.9226 | 0.973767 | 0.5907 | 0.972376 | 0.6060 |
| **PT** (°) | 0.992294 | 0.9526 | 0.989582 | 0.8510 | 0.986430 | 0.7167 | 0.976826 | 0.6870 | 0.966689 | 0.4530 |
| **PI** (°) | 0.983932 | 0.5299 | 0.980491 | 0.3745 | 0.984433 | 0.6089 | 0.951754 | 0.1497 | 0.938120 | 0.0810 |
| **PTh** (mm) | 0.989832 | 0.8574 | 0.992459 | 0.9592 | 0.983503 | 0.5600 | 0.948990 | 0.1243 | 0.979270 | 0.8058 |
| **S3DL** (mm) | 0.976192 | 0.2181 | 0.979233 | 0.3244 | 0.963051 | 0.0557 | 0.877838 | **0.0015** | 0.969463 | 0.5246 |

The Shapiro-Wilk W test with a p-value < 0.05 indicates that the data deviates from a normal distribution (**bold character**).
