# Supplementary material for: Normality of sagittal spinal alignment parameters reveals evolutionary signals in healthy adults across five countries
Source: Sci Rep. 2025 Oct 10;15:35484. doi: 10.1038/s41598-025-19366-z (PMC12514220; doi:10.1038/s41598-025-19366-z)
Supplement: Supplementary file 3 — Supplementary Material 3 [file 41598_2025_19366_MOESM3_ESM.docx]

**Supplementary Table 3. Difference in normality of alignment between age groups**

|  | **Age < 30 yeas old** | | **Age > 30 yeas old** | |
| --- | --- | --- | --- | --- |
| **Parameters** | **Shapiro-Wilk W test** | | **Shapiro-Wilk W test** | |
|  | **W value** | **p value** | **W value** | **p value** |
| **CL** (°) | 0.983764 | 0.0892 | 0.985075 | 0.2182 |
| **TK** (°) | 0.996407 | 0.9805 | 0.989567 | 0.5092 |
| **LL** (°) | 0.991642 | 0.5618 | 0.988211 | 0.4011 |
| **LL1-4** (°) | 0.982596 | 0.0664 | 0.992886 | 0.8106 |
| **LL4-S (°)** | 0.985759 | 0.1470 | 0.987168 | 0.3299 |
| **SS** (°) | 0.974472 | **0.0089** | 0.989758 | 0.5257 |
| **PT** (°) | 0.996768 | 0.9894 | 0.980413 | 0.0827 |
| **PI** (°) | 0.971916 | **0.0049** | 0.982802 | 0.1366 |
| **PTh** (mm) | 0.985385 | 0.1339 | 0.984439 | 0.1916 |
| **S3DL** (mm) | 0.970147 | **0.0032** | 0.990275 | 0.5715 |

The Shapiro-Wilk W test with a p-value < 0.05 indicates that the data deviates from a normal distribution (**bold character**).
